# Osteoclast Fusion is Based on Heterogeneity Between Fusion Partners

**DOI:** 10.1007/s00223-014-9864-5

**Published:** 2014-05-27

**Authors:** Anne-Sofie Hobolt-Pedersen, Jean-Marie Delaissé, Kent Søe

**Affiliations:** Clinical Cell Biology, Vejle Hospital/Lillebaelt Hospital, Institute of Regional Health Research, University of Southern Denmark, Kabbeltoft 25, 7100 Vejle, Denmark

**Keywords:** Osteoclast fusion, Heterogeneity, CD47, DC-STAMP, Syncytin-1, Connexin 43

## Abstract

Bone-resorbing osteoclasts are formed through fusion of mononucleated precursors. Their choice of partners during the fusion process remains unclear. We hypothesized that osteoclasts are selective in their choice of fusion partner and that this selectivity is based on heterogeneity among the cells with respect to their maturation stage and their expression and cellular organization of fusion factors. Support for this hypothesis was found from immunofluorescence staining of the osteoclast fusion factors CD47, dendritic cell-specific transmembrane protein (DC-STAMP), and syncytin-1. These stainings revealed heterogeneous localization patterns of all three factors within a given culture of osteoclasts. CD47 was found to be localized primarily in small osteoclasts and preosteoclasts, which were also positive for DC-STAMP but negative for cathepsin K expression. A role of CD47 in the early osteoclast fusion steps was also suggested from experiments with a CD47 blocking antibody, which resulted in an inhibition of the fusion of small osteoclasts. Conversely, blocking of connexin 43 affected the fusion of larger osteoclasts with four or more nuclei. The suggestion that different fusion factors function at different stages of osteoclast fusion supports the idea of heterogeneity in the osteoclast population; our results suggest that osteoclast fusion is indeed based on heterogeneity. Considering the in vivo environment in which osteoclasts develop and fuse, our findings seem very applicable and provide novel, important insight into key issues in bone and fusion research.

## Introduction

Osteoclasts (OCs) are large multinucleated cells formed through fusion of mononucleated precursors of monocytic origin. OCs are responsible for resorption of bone during the natural maintenance of bone homeostasis, and their formation is crucial for human health. In general, fusion between cells is a common biological phenomenon that is fundamental in events like the fusion of sperm and oocyte during fertilization and the multiple fusions of trophoblasts in the placenta. Important fusion events are also the fusion of myoblasts into muscle fibers and that of macrophages to produce giant cells at chronic inflammatory sites [1–3]. Much effort has been devoted to identify the factors involved in these different kinds of fusion, and some proteins have been shown to be common to several of the fusion processes studied. One of these proteins is the endogenous retroviral-derived protein syncytin-1. The binding of this protein to its receptor, alanine-, serine-, cysteine-preferring neutral amino acid transporter 2 (ASCT2), gives rise to conformational changes in syncytin-1 that pull the lipid bilayers together and cause the cells to fuse [4]. Syncytin-1 is involved in human OC fusion [5] and human trophoblast fusion [6, 7], and it has also been suggested to play a role in the fusion of human myoblasts [8].

Another well-known fusion factor is CD47, which is an ubiquitously expressed glycoprotein that interacts with signal-regulatory protein alpha (SIRPα) as part of the multinucleation process in both OCs and macrophages [9–11]. The interaction between CD47 and SIRPα, which are both members of the superfamily of immunoglobulins, also plays a role in the immunological recognition of “self” to prevent macrophage phagocytosis [12]. This property could make macrophages distinguish potential fusion partners from matters to phagocytize, as previously suggested [13, 14]. Like CD47, dendritic cell-specific transmembrane protein (DC-STAMP) is another important fusion-related factor that has been demonstrated to be important for the fusion of cells of monocytic origin [15–17]. DC-STAMP is described as a central fusion mediator in OCs because a variety of factors can regulate OC fusion by affecting DC-STAMP expression [18]. Interestingly, it has been demonstrated that in order for OC formation to take place, the expression of DC-STAMP is only required in one of two fusing partners [16]. It is noticeable that there is significant diversity among the recognized fusion factors, and the factor connexin 43 (Cx43) is unique in regard to function compared to syncytin-1, CD47, and DC-STAMP. Cx43 forms gap junctions, which allows intercellular communication that has previously been shown to be important to permit fusion of both trophoblasts, myoblasts, and OCs [19–23].

Several studies have provided important details about OC fusion factors and their importance to the fusion process. This research has mainly been focused on the expression of genes encoding potential fusion factors, in vitro blocking, or overexpression [9, 10, 15, 19, 20]. In other cases, knockout animal models were used [9, 11, 16, 17, 24, 25]. Common to these studies is that the obtained results reflect the response of the OC population as a whole in relation to the total gene expression, the total change in OC number, or the end-point effects on bone phenotype. Less emphasis has been placed on how cells like OCs behave during a fusion event. It is clear that fusion is a multistep process in which cells recognize and migrate toward each other, then come into close proximity and eventually fuse. However, how do the cells select their fusion partners? Is some kind of selectivity involved in this action? These questions naturally arise because understanding the preferences and behaviors of cells during fusion is of biological and medical interest.

Mensah et al. [26] suggested that fusion among preosteoclasts (preOCs) is determined by cell-population heterogeneity in relation to the presence or the absence of DC-STAMP on the extracellular membrane. Likewise, a pattern of complementarity regarding the location of syncytin-1 was observed in OCs that were in close contact in phagocytic cups where syncytin-1 was found to be up-concentrated at the area of the membrane facing the fusion partner [5]. Together, this recent research suggests that heterogeneity does indeed exist in populations of fusing (pre)OCs.^1^ These studies also imply that heterogeneity may not necessarily be regulated only by gene expression or protein synthesis but also by the organization and localization of proteins. However, further research on this topic is required to improve the general understanding of cell fusion.

We therefore designed a new study to test the hypothesis that fusion of (pre)OCs is based on selectivity in the choice of fusion partner within a heterogeneous population. We used human (pre)OCs differentiated from CD14^+^ peripheral blood cells and studied the characteristics of the described fusion factors in order to reveal differences in their function and localization. From our findings, we propose that (pre)OCs are not just fusing at random but that they specifically select their fusion partners on the basis of complementarities.

## Materials and Methods

### Antibodies and Visualization of F-actin

The primary antibody mouse anti-human CD47 (clone B6H12; BD, Franklin Lakes, NJ, USA) was used for both functional blocking experiments and immunofluorescence (IF) staining. For the control, we used mouse IgG1 isotype control (BD). The following additional primary antibodies were used for IF: mouse anti-human syncytin-1 clone 7E3 (a generous gift from Professor Lars-Inge Larsson and Bolette Bjerregaard, PhD), rabbit anti-human DC-STAMP (polyclonal C-17; Santa Cruz Biotechnology Inc., Santa Cruz, CA, USA) and goat anti-human CathepsinK (CatK, polyclonal N-20; Santa Cruz Biotechnology Inc.). Secondary antibodies were as follows: Alexa Fluor 568 conjugated: goat anti-mouse, goat anti-rabbit, and donkey anti-mouse, as well as Alexa Fluor 488 conjugated: rabbit anti-goat and goat anti-rabbit (Invitrogen, Carlsbad, CA, USA). Alexa Fluor 488 and 647 phalloidin (Invitrogen) were used for visualization of F-actin.

### Cell Culture

CD14^+^ monocytes were purified from blood of human donors (approved by the local ethical committee, 2007-0019) by centrifugation though Ficoll (Amersham, GE Healthcare, Little Chalfont, United Kingdom) and subsequent immunomagnetic isolation with MagCellect Streptavidin Ferrofluid (R&D Systems, Minneapolis, MN, USA). A more detailed experimental description has been published previously [27]. Cells were cultured at 37 °C in 5 % CO_2_, supplied with αMEM (Invitrogen) containing 10 % fetal calf serum (FCS) (Biological Industries, Kibbutz Beit-Haemek, Israel) and 25 ng/ml macrophage colony-stimulating factor (M-CSF, R&D Systems). After a 2-day culture period, the medium with FCS and M-CSF was refreshed, and 25 ng/ml human receptor activator of nuclear factor kappa-B ligand (RANKL, R&D Systems) was added. The cells were subsequently seeded in either 8-well Lab-Tek chamber slides (Nunc, Roskilde, Denmark) or 96-well plates (Greiner, Frickenhausen, Germany) depending on the assay. Here we define preOCs as mononucleated CD14^+^ cells, and OCs are defined as multinucleated cells differentiated from preOCs with M-CSF and RANKL, as described above.

### Functional Blocking

The cells were seeded in 96-well plates (7.5 × 10^4^ cells per well). After 2 days with M-CSF followed by 3 days with both M-CSF and RANKL, the preOCs reached a very early fusion stage (as identified by light microscopy). They were then supplied with fresh medium containing M-CSF, RANKL, and either one of the inhibitors or a corresponding control supplement. The cells were subsequently cultured for another 4 days. In the CD47 blocking experiment, the preOCs were cultured with either 10 μg/ml CD47 antibody, 10 μg/ml mouse IgG1 isotype control antibody, or without any additive. The medium with these supplements was renewed every second day. In the Cx43-blocking experiment, the preOCs were cultured with 100 μM 18α-glycyrrhetinic acid (18α-GA, Sigma-Aldrich, St. Louis, MO, USA) dissolved in DMSO, or with the corresponding volume of DMSO (0.2 %). The medium was renewed daily with fresh conditioned medium (from a parallel running (pre)OC culture) supplemented with 18α-GA or DMSO. After 4 days of culture with inhibitors, the cells were fixed and stained with Giemsa and May-Grünwald as previously described [28]. By analysis on an Axiovert 200 microscope (Carl Zeiss, Oberkochen, Germany), all the multinucleated OCs and their number of nuclei were counted systematically in every second counting field in three replicate culture wells for each treatment.

### IF Staining

PreOCs were seeded in 8-well chamber slides (5–10 × 10^4^ cells per well depending on the assay), and the medium with RANKL was renewed after 3 days. After incubation with RANKL for 5 days, the OCs were fixed and stained with the CD47, DC-STAMP, or syncytin-1 antibodies listed above and phalloidin, as previously described [5]; however, in this case, the incubation time with primary antibody was 60 min. If CD47-DC-STAMP or CD47-CatK double stainings were performed, the cells were washed, incubated with each of the primary antibodies consecutively, washed again, and then incubated with both secondary antibodies simultaneously. Nuclei were visualized by mounting the cells in ProLong Gold with DAPI (Invitrogen), and images were obtained systematically with an Axio Imager Z1 microscope (Carl Zeiss) with Isis software, version 5.3.1 (Metatsystems, Altlussheim, Germany). The confocal analysis was performed on an Olympus Fluoview FV10i microscope (Olympus Corporation, Shinjuku, Tokyo, Japan), and images were processed with Imaris version 7.3.1 (Bitplane AG, Zurich, Switzerland).

### Software, Data Analyses, and Statistics

The area measurement of CD47 immunostained (pre)OCs on the obtained images was performed by ImageJ software (National Institutes of Health, Bethesda, MD, USA). For this measurement, two categories of (pre)OCs were defined according to their CD47 status. The category of CD47-positive cells included all (pre)OCs with CD47 in the whole cell or concentrated in specific areas at the membrane, while the negative category contained the CD47-negative (pre)OCs and those with CD47 localized only around the nuclei, because CD47 localized in this area is expected to be insignificant for the fusion process. The areas measured were defined as the two-dimensional areas of the cells as measured from above. In case of the CD47-DC-STAMP and CD47-CatK double stainings, images were obtained, and all the cells were systematically evaluated for their status in regard to surface localization of CD47 and DC-STAMP and their expression of CatK. All graphs and statistics were performed by GraphPad Prism software, version 4 (GraphPad Software, San Diego, CA, USA), and statistical significance was defined as *P* < 0.05. The final figures were composed using CorelDraw X3 (Corel Corporation, Ottawa, Ontario, Canada).

## Results

### Blocking of Fusion Factors Affects Different Stages in OC Fusion

**Fig. 1:**
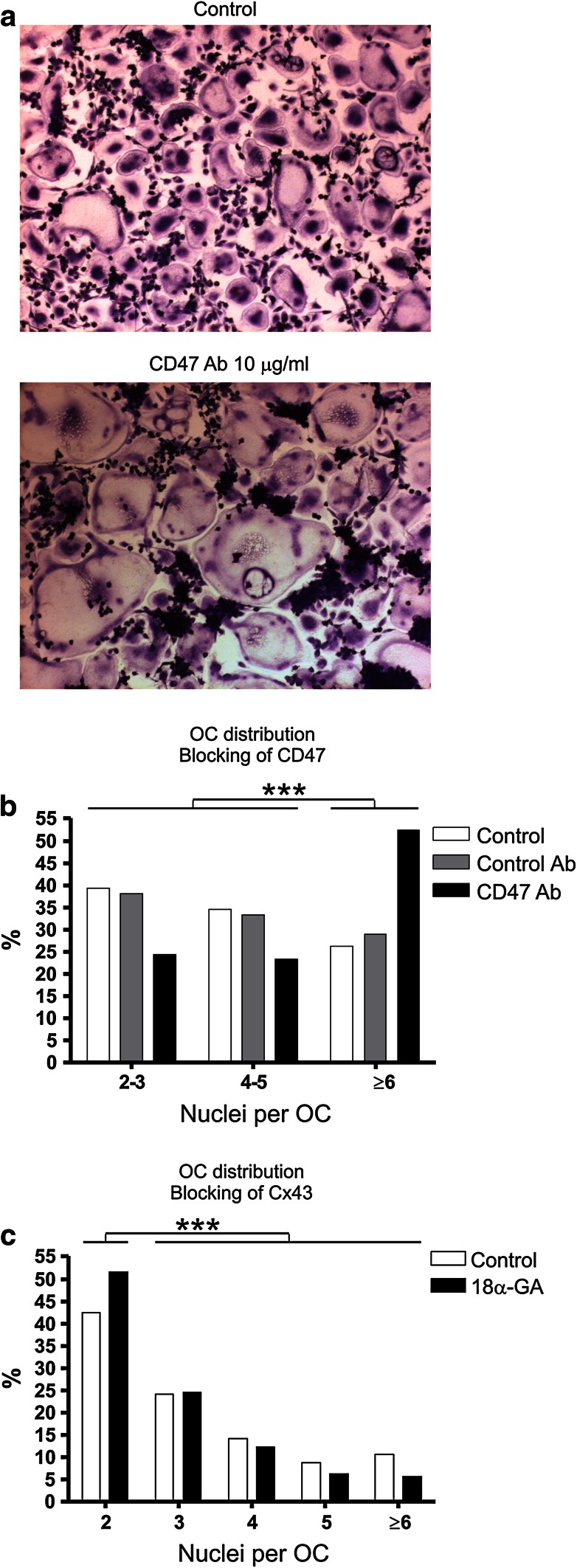
Blocking of fusion factors CD47 and Cx43 affects fusion of OCs at different fusion stages. **a** Light microscopy images, at equal magnification, of Giemsa-stained (pre)OC cultures incubated without additives or with CD47-blocking antibody. **b** Relative distribution of OCs according to treatment and their number of nuclei. For each culture condition, counts from 3 different cultures were pooled, ranked according to the number of nuclei per OC, and the percentages of OCs with a given number of nuclei calculated. Fisher’s exact test of the relation between culture condition and proportion of OCs with 6 or more nuclei compared to those with fewer nuclei: CD47 antibody–treated OCs versus both of the controls, *P* < 0.0001 in both tests; isotype control vs. control without additives, *P* = not significant. **c** Relative distribution of OCs in an 18α-GA-treated (pre)OC culture and control according to their number of nuclei. Quantifications were done as in **b**. Fisher’s exact test of the relation between culture condition and proportion of OCs with 2 nuclei compared to those with 3 or more nuclei: 18α-GA-treated OCs vs. control, *P* = 0.0007

To evaluate the function of CD47 and Cx43 in human OC fusion, CD47 and Cx43 inhibitors were added to cultures of preOC at an early differentiation stage, at which time fusion had just started. Incubation of the preOCs with CD47-blocking antibody resulted in an increased formation of large nuclei-rich OCs, which were easily identified by light microscopy (Fig. 1a). This finding was consistent in four experiments. A comparison of the OCs treated with CD47 antibody with those of control cultures revealed an interesting distribution of the number of nuclei per OC: the proportion of OCs with six or more nuclei was significantly different from the proportion of OCs with fewer nuclei. While 26 and 29 % of the OCs in the control cultures had six or more nuclei, this proportion reached 52 % of the OCs treated with CD47 antibody (Fig. 1b).

To examine the role of Cx43 in OC fusion, we used the inhibitor 18α-GA and compared the treated OCs with those of a corresponding control culture. When fusing OCs were treated with this inhibitor, significantly fewer of the large OCs were formed, as opposed to an overweight of the small binucleated OCs (Fig. 1c). These analyses show that both 18α-GA and CD47 antibody have a fusion-stage-dependent effect on OCs. However, while blocking of CD47 seemed to favor the formation of large OCs and to reduce the formation of the smaller OCs (Fig. 1b), the Cx43 blockage had the opposite effect: it inhibited the formation of OCs with four or more nuclei in favor of that of smaller OCs (Fig. 1c). Our findings suggest heterogeneity in the population of fusing (pre)OCs. We therefore investigated this further by analyzing the presence of CD47 in such cells with the purpose of revealing any heterogeneity in its localization.

### CD47-presenting Cells have a Smaller Surface Area

**Fig. 2:**
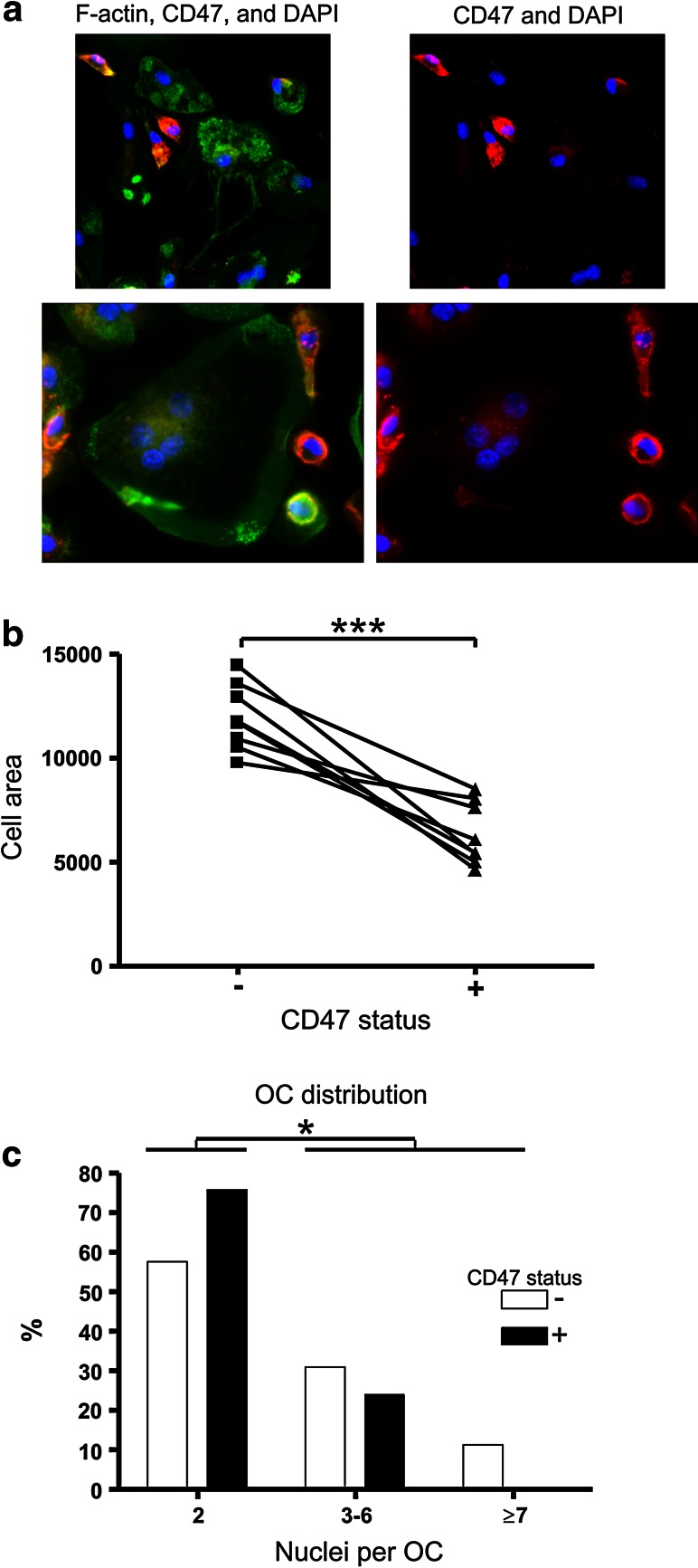
CD47 is preferentially found in (pre)OCs, which are small both with respect to size and number of nuclei. **a** Staining of CD47 (*red*) and F-actin (*green*) in (pre)OCs differentiated for 5 days with RANKL. Nuclei were visualized with DAPI (*blue*). **b** The cells (seeding density: 5 × 10^4^ per well) were assed for CD47 status. Then the area of each cell in 8 culture wells was measured in square pixels, and the sizes of the CD47-positive and CD47-negative cells were compared. Paired *t* test, *P* = 0.0004. **c** Relative distribution of CD47-positive and CD47-negative OCs according to their number of nuclei was calculated as described under Fig. 1 **b**. Fisher’s exact test of the relation between CD47 status and proportion of OCs with 2 nuclei compared to those with 3 or more, *P* = 0.0209

To visualize the presence of CD47 in fusing (pre)OCs in culture, and to supplement the results obtained by the blocking of CD47, IF staining of CD47 and F-actin was performed. Cells were fixed and stained at a point in time at which fusion was ongoing and where both preOCs and OCs at various fusion stages were observed. The staining showed the presence of CD47 both in preOCs and OCs; however, the most striking finding was that the cells presenting CD47, either in the whole cell or at the cell membrane, appeared to be relatively small (Fig. 2a). Many of these CD47-positive cells were rather compact, whereas the large OCs with a widespread flat appearance were often CD47 negative. In order to quantify the relevance of this indicative finding, (pre)OCs were categorized according to their CD47 status. A measurement of the area of (pre)OCs positive for CD47 revealed that they had a significantly smaller area than the CD47-negative cells (Fig. 2b). In support of this finding, we also discovered that CD47-positive OCs had significantly fewer nuclei than the CD47-negative OCs (Fig. 2c). Together, these results indicate that the CD47-presenting (pre)OCs are smaller than the CD47-negative cells in terms of both their size and their number of nuclei.

### CD47 is Presented Early During OC Differentiation

The results displayed in Fig. 2 suggest that CD47 is primarily found in small OCs with few nuclei.

**Fig. 3:**
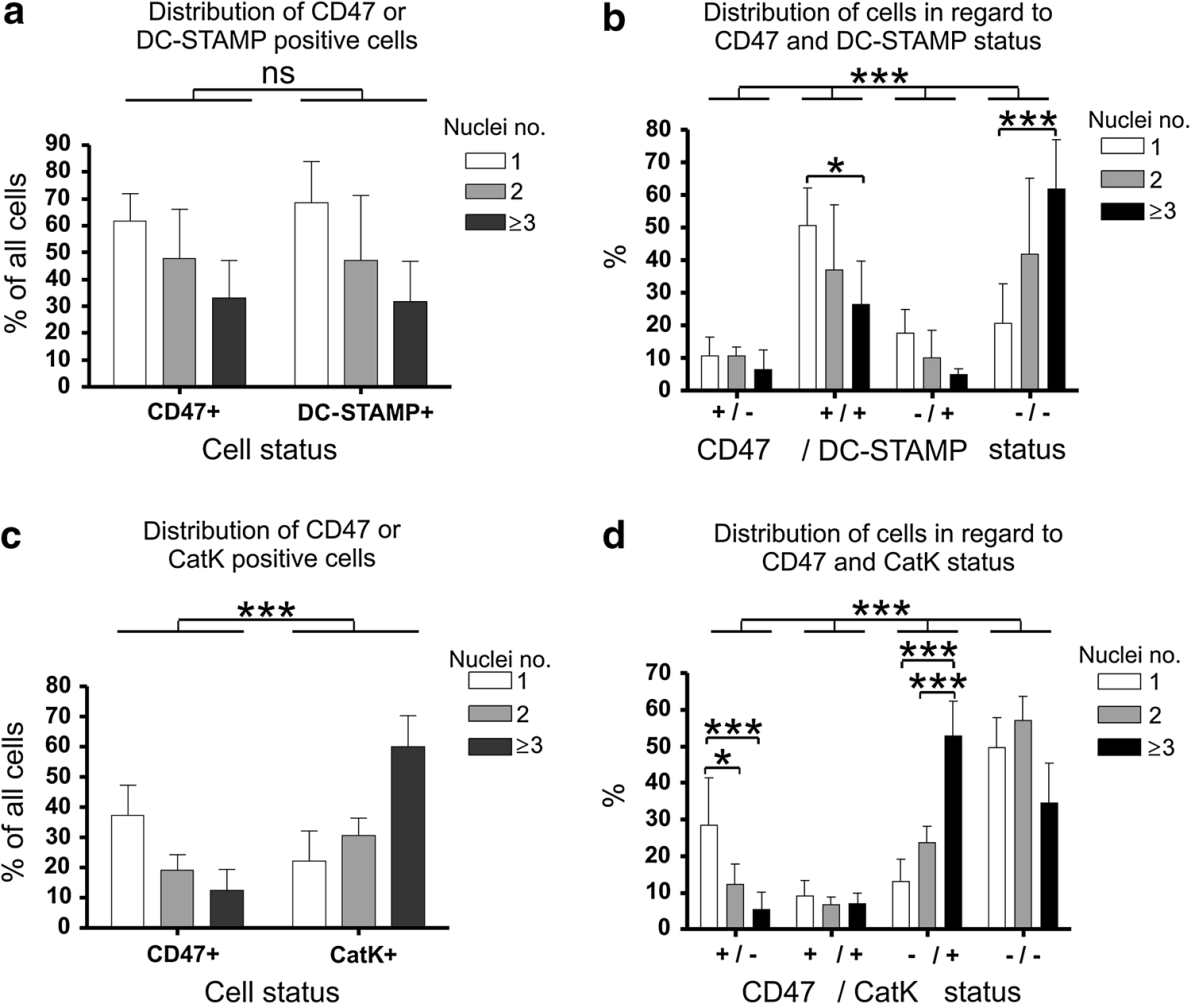
Similar to the OC marker DC-STAMP, surface localization of CD47 is primarily found among preOCs and OCs at early differentiation stages (seeding density: 1 × 10^5^ per well). **a** Distribution of CD47- or DC-STAMP-positive (pre)OCs according to their number of nuclei. Counts of (pre)OCs from 4 different CD47 and DC-STAMP double-stained cultures were pooled, ranked according to the number of nuclei per cell, and percentages calculated of (pre)OCs, with a given number of nuclei, presenting CD47 or DC-STAMP in the cellular membrane. Statistics: 2-way ANOVA, *P* = not significant. **b** Distribution of all (pre)OCs analyzed according to their CD47 and DC-STAMP status and their number of nuclei. Statistics: 2-way ANOVA test of correlation between nuclei distribution and cell status among 4 variable CD47/DC-STAMP cell phenotypes (±), (+/+), (∓), and (−/−), *P* < 0.0001. **c** Distribution of CD47- or CatK-positive (pre)OCs according to their number of nuclei. Data were pooled from countings of (pre)OCs in 4 different CD47 and CatK double-stained cultures. Cells were ranked according to their number of nuclei and percentages calculated of CD47- or CatK-positive cells. Statistics: 2-way ANOVA, *P* = 0.0005. **d** Distribution of all (pre)OCs analyzed according to their CD47 and CatK status and their number of nuclei. Statistics: 2-way ANOVA test of the correlation between nuclei distribution and cell status among the 4 variable CD47/CatK cell phenotypes (±), (+/+), (∓), and (−/−), *P* < 0.0001. Bonferroni posttests: statistical significance was defined as *P < 0.05, and ***P < 0.001

To relate these findings to OC maturity in terms of differentiation stage, we correlated the presence of CD47 to that of OC differentiation markers DC-STAMP and CatK in the individual (pre)OCs. Membrane-associated DC-STAMP is well documented to be a marker of early OC differentiation stages [26, 29], while CatK is a marker for rather late stages [30]. We consider multinucleation of the OCs to be an indicator of progressed differentiation, and thereby we regard cells with several nuclei as more mature than those with few. The (pre)OCs were analyzed for their membrane localization of CD47 and DC-STAMP (Fig. 3a, b), or their CD47 status was compared to CatK expression (Fig. 3c, d). Analyses were done for each individual cell using the described categorization. The results revealed that the cells show comparable patterns of CD47 and DC-STAMP presentation, with the highest prevalence of both proteins in preOCs. The proportion of cells presenting CD47 or DC-STAMP at their membrane declined among the binucleated OCs and further among those with three or more nuclei (Fig. 3a). Consistently, double staining of CD47 and DC-STAMP showed that these factors are primarily presented by the same (pre)OCs (Fig. 3b). Actually, 77 % of the CD47-positive (pre)OCs were also positive for surface DC-STAMP (data not shown). On the contrary, the corresponding comparison between CD47 and CatK in the cells showed significantly different patterns of (pre)OCs distribution according to number of nuclei. Because the presence of CatK in the cells became more frequent with their differentiation, the proportions of CatK presenting (pre)OCs reached the highest level among those cells with three or more nuclei (Fig. 3c). Consistently, only a little overlap between CD47 and CatK presentation in the individual (pre)OCs was found (Fig. 3d). Less than 28 % of all CD47-positive cells in the analysis showed concurrent expression of CatK (data not shown). Thus, the pattern of CD47 presence among the (pre)OCs closely resembled that described for their surface expression of DC-STAMP, and thereby CD47 is characteristically related to (pre)OCs early in their differentiation.

### CD47 is Localized at the Interface Between Fusing (pre)OCs

**Fig. 4:**
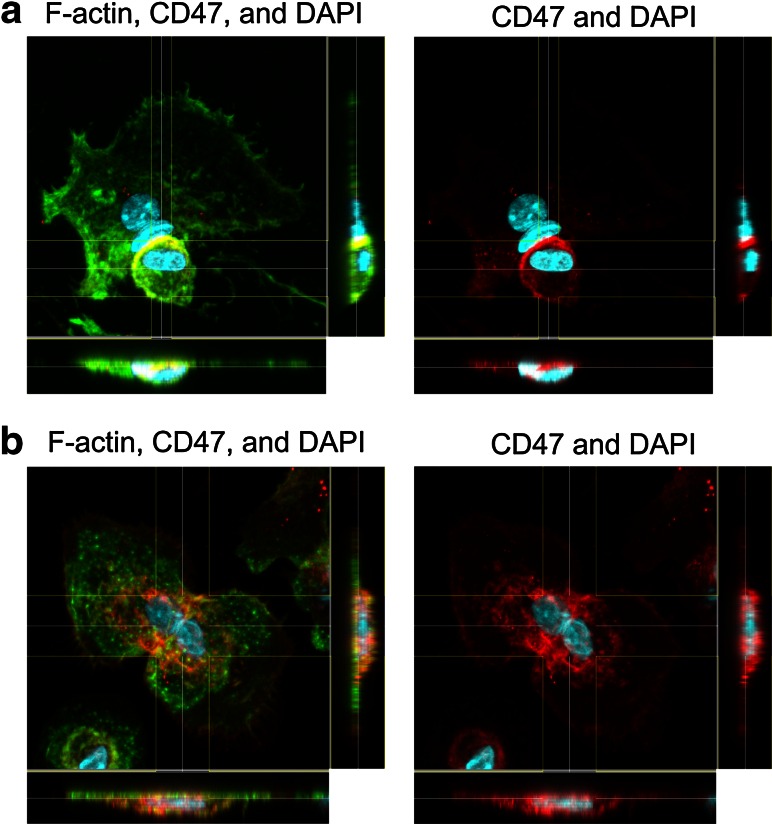
Visualization by confocal microscopy of CD47 in fusing (pre)OCs. Staining of CD47 (*red*) and F-actin (*green*) in (pre)OCs (1 × 10^5^ per well) differentiated for 5 days with RANKL. Nuclei were visualized with DAPI (*blue*). *Z* stacks along the *x*- and *y*-axes are presented at bottom and on *right side* of images. **a** CD47 is localized to the part of the cell membrane where a preOC is tightly interacting with a binucleated OC. **b** Two preOCs in close proximity. Both show strong CD47 signals at the part of their membrane facing the other cell

The use of confocal microscopy revealed in detail the localization of CD47 in fusing (pre)OCs. Figure 4 shows (pre)OCs interacting in close proximity, which strongly indicates forthcoming fusion. In the examples shown in Fig. 4a, b, CD47 was clearly concentrated at the part of the preOC membrane facing the fusion partner in Fig. 4a in the smaller of the two partners and in Fig. 4b in both partners. From the cross-sectional representation in Fig. 4a, a clear front of CD47 is revealed at the part of the preOC cell membrane that is in contact with the fusion partner.

### Heterogeneity in the Localization of the Fusion Factors DC-STAMP and Syncytin-1

**Fig. 5:**
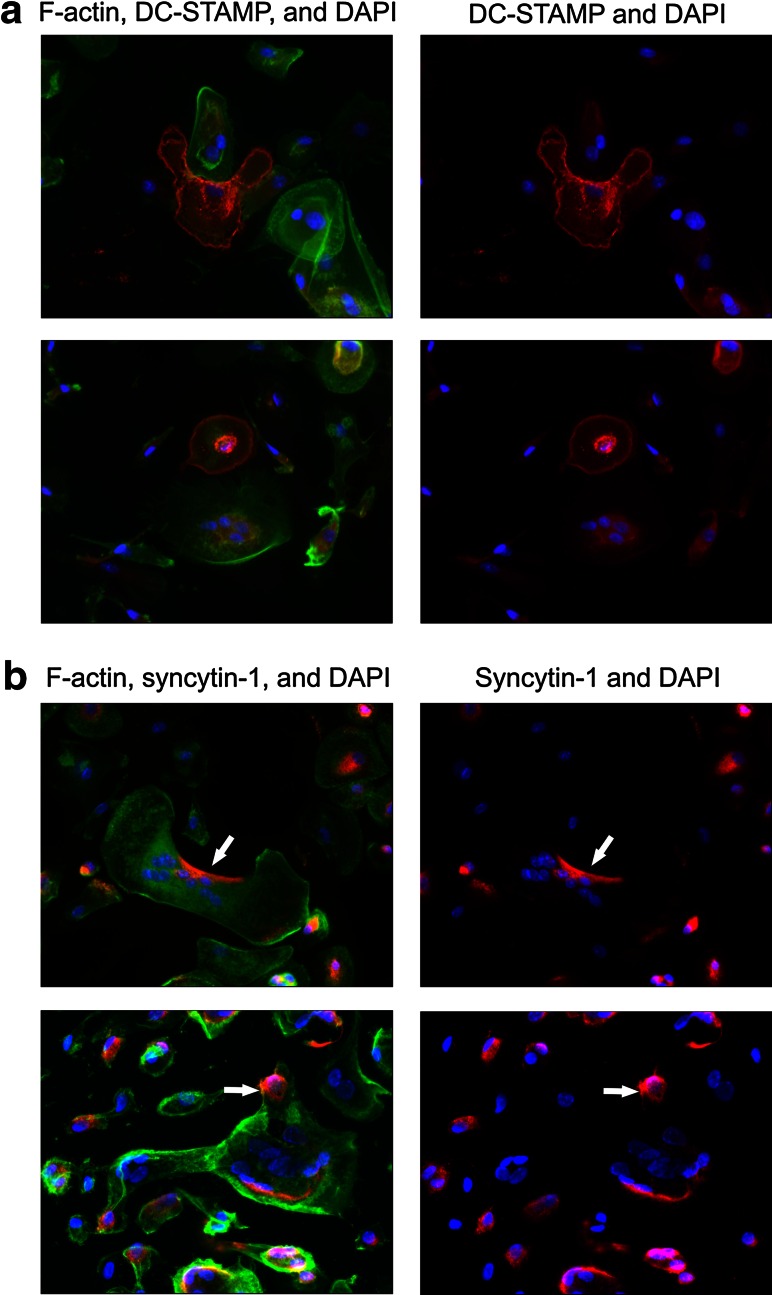
DC-STAMP and syncytin-1 are heterogeneously localized in (pre)OCs. Visualization of DC-STAMP and syncytin-1 in (pre)OCs (1 × 10^5^ per well) differentiated for 5 days with RANKL. Nuclei were visualized with DAPI (*blue*). **a** Staining of DC-STAMP (*red*) and F-actin (*green*). **b** Staining of syncytin-1 (*red*) and F-actin (*green*). Note pattern of heterogeneous localization of both these proteins in cells in close proximity and opposite polarization of syncytin-1 (*arrows*)

The heterogeneity in the presence of CD47 among cells at different differentiation stages in (pre)OC culture and the clear presentation of this protein at the interface of fusing preOCs support the idea that CD47 is an important factor in OC formation (Figs. 2, 3, 4). These findings led to related studies of the localization of other known OC fusion factors. For this purpose, the factors DC-STAMP and syncytin-1 were chosen because of their documented involvement in OC fusion [5, 15–17]. Interestingly, heterogeneity was also found in the expression and localization of these factors. IF staining of F-actin, together with either DC-STAMP or syncytin-1, revealed complementary protein localization patterns in our (pre)OC cultures in both cases. Several of the DC-STAMP-stained cells exerted a pattern of either absence or of clear presence of this protein in the cellular membrane. The results in Fig. 5a show examples of such DC-STAMP-positive and -negative cells. The top pictures illustrate two cells in close proximity. The DC-STAMP-positive partner has a conformation resembling a phagocytic cup indicative of fusion [5]. Likewise, staining of syncytin-1 showed heterogeneity in the localization of this protein. In this case, opposite polarization of syncytin-1 in two fusion partners was observed in several cells (Fig. 5b), which supports our previously published findings [5]. Collectively, these results indicate a pattern of diversity in the localization of several known fusion factors between individual cells in a (pre)OC culture.

## Discussion

The aim of the present study was to explore how OCs choose their fusion partners—a topic that until now has received little attention. We hypothesized that heterogeneity among (pre)OCs plays a role in their selection of fusion partners. Through functional blocking of CD47 and Cx43, it was suggested that OC fusion is differently affected depending on the fusion stage of the cells. This indicated that not all (pre)OCs express CD47, which could be confirmed through double IF staining of CD47 and DC-STAMP (early marker) or CatK (late marker). These stainings showed that CD47 is preferentially found at early stages of OC differentiation and they thereby match the results obtained by functional blocking of CD47, which selectively reduced the number of OCs with few nuclei. Another indication of heterogeneity among the (pre)OCs was found in form of complementarity in the localization of both syncytin-1 and DC-STAMP in potential fusion partners. On the basis of these results, we suggest that OC fusion is based on heterogeneity between fusion partners.

Although our proposal for selectivity in OC fusion depending on the differentiation stage of the cells is new, it is supported by the literature. Mensah et al. [26] reported that RANKL induces a mixed population of murine preOCs with either high or low surface expression of DC-STAMP and demonstrated that only in a co-culture of these two cell populations did efficient fusion take place. Chiu et al. [29] found that DC-STAMP was differentially expressed in human OC cultures, and it was reported that OCs express DC-STAMP early during differentiation but lose it when they mature and become multinucleated, similar to what we here report for CD47. This highlights that a known fusion-related factor is differentially expressed in OC cultures and that this favors fusion. We also show here that DC-STAMP is preferentially expressed in early (pre)OCs (Fig. 3a,b), which may also contribute to heterogeneity between potential fusion partners (Fig. 5a). Similarly, we previously reported that syncytin-1 was frequently concentrated at the part of the cell membrane where (pre)OCs were facing their possible partners [5]. This is further supported by our results here (Fig. 5b).

In addition to the syncytin-1 and DC-STAMP data, we also found heterogeneity in terms of the presence or absence of CD47 on the cell membrane of (pre)OCs. IF staining showed that CD47 is primarily present in those cells that are small, both in regard to area and number of nuclei (Figs. 2, 3). Furthermore, CD47 presence on the cell membrane was found to positively correlate with the presence of DC-STAMP early in the OC differentiation process (Fig. 3b), while it correlated negatively with CatK (Fig. 3d). This result is supported by a previous report showing that CD47 was mainly expressed in mononucleated macrophages, while it was down-regulated along with multinucleation [10]. Taken together, these data highlight the notion that CD47 is primarily expressed in less differentiated (pre)OCs with few nuclei. This discovery may be the key to understand the results of our CD47-blocking experiment shown in Fig. 1, in which we found that blocking of CD47 inhibited the generation of OCs with fewer than six nuclei but favored the formation of large OCs with six nuclei or more. At a first glance, we found these results surprising because it was previously reported that blocking or knocking out CD47 reduced fusion in both mouse OC and rat macrophage cultures [9–11, 24].

However, in this context, it is important to consider the elementary differences between our experimental approach and those used in the aforementioned studies, as follows. (1) The published data have been generated from studies based on murine models both in vivo and in vitro. It is indeed possible that the apparent lack of consistency between our results and those available in the literature may be explained by differences between human and murine OCs in general and their culture conditions in particular. It is in this aspect important to emphasize that our cultures consist of purified CD14^+^ cells from peripheral blood, while the studies on mouse cells were done using unpurified bone marrow cultures. Such bone marrow cultures do not only contain (pre)OCs but also other cells. (2) The results obtained from CD47 knockout mice are not consistent because Uluckan et al. [24] and Maile et al. [11] did not find a decreased number of OCs in vivo, whereas Lundberg et al. [9] did. (3) Previous studies were exclusively based on analyses of the number of multinucleated OCs (three or more nuclei) or the average number of nuclei per OC. When OCs with two nuclei are excluded from such an analysis, the very first fusion events is missed—and thereby also valuable information when investigating effects on fusion. This may indeed influence the results and could, for example, mask specific details concerning the fusion stage of the cells affected. In the present study, we made a detailed analysis of the distribution of OCs according to their number of nuclei (Fig. 1b), which has not been reported before. (4) It is important to stress that we did indeed observe an inhibition of OC fusion using the CD47 antibody, although this was only observed for small OCs. It is possible that the antibody shifts the balance of the fusion process because the small CD47-positive cells are prevented from fusing. The CD47-negative (pre)OCs may therefore be forced to fuse with other CD47-negative cells instead (which generally have more nuclei) (Figs. 2, 3). This results in an increased number of OCs with many nuclei, although the reason for this was an inhibition. (5) The addition of CD47 antibody was introduced already at the beginning of the culture in the study of Lundberg et al. [9], whereas we added it after 3 days with RANKL at the onset of fusion. This may also affect the outcome of the experiments differently.

On the basis of these issues, we conclude that heterogeneity in CD47 status between human (pre)OC plays an important role in the selection of OC fusion partners. In contrast to CD47, Cx43 appeared primarily to be involved in the fusion of OCs with several nuclei. The addition of the gap junction inhibitor 18α-GA to our (pre)OC cultures resulted in a high proportion of small OCs, while the formation of OCs with four or more nuclei was inhibited (Fig. 1c). Kylmäoja et al. [23] also used 18α-GA to inhibit the formation of gap junctions in RAW 264.7- and mouse bone marrow-derived cells seeded on bone slices. In support of our findings, they found a dose-dependent decrease in the number of large, nuclei-rich OCs in the presence of 18α-GA. Other studies have also shown a role of gap junctions in OC fusion [19, 20]. In summary, CD47 and Cx43 appear to affect fusion differently, and both support the importance of heterogeneity in OC fusion.

Parallels may be drawn from our in vitro findings of heterogeneity to physiological settings, where OC fusion also takes place between cells with different characteristics. Spatially distinct environments with different local stimuli exist on the bone surface and in the bone marrow, and the properties of the cells change as they move toward and reach the bone surface where fusion takes place [31]. In general, heterogeneity between fusion partners is broadly recognized and also essential in other biological fusion processes [1–3]: cytotrophoblasts fuse with the syncytiotrophoblast and show heterogeneous expression of fusion factors [32–34], and myoblasts and myotubes that fuse in muscle tissue show different characteristics both in relation to motility and protein expression [35–37]. This highlights the physiological relevance of our results.

In conclusion, we have shown that OC fusion is based on heterogeneity between fusion partners. This emerging concept may open new doors when exploring the enigma of OC fusion.

## Footnotes

1 (Pre)OCs refers to a population of cells that consists of mixture of preOCs and OCs.
